# Supplementary material for: Ground-dwelling arthropods of pinyon-juniper woodlands: Arthropod community patterns are driven by climate and overall plant productivity, not host tree species
Source: PLoS One. 2020 Aug 26;15(8):e0238219. doi: 10.1371/journal.pone.0238219 (PMC7449382; doi:10.1371/journal.pone.0238219)
Supplement: S2 File — (DOCX) [file pone.0238219.s010.docx]

|  | Transformation | Distance | Stress | Goodness of fit test for each sample (*P*) |
| --- | --- | --- | --- | --- |
| Ants | None | Jaccard | 0.212 | all less than 0.04 |
| Beetles | Square Root | Bray | 0.184 | all less than 0.03 |

**S4 Table 1.** Ordination details and fit metrics for ant and beetle communities.


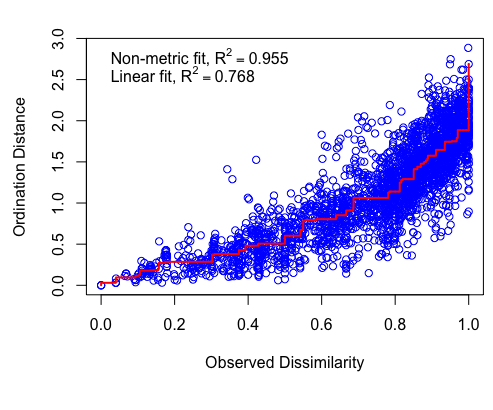


**S4 Fig. 1.** Shepard diagram of ordination fit for ant dissimilarity matrix.


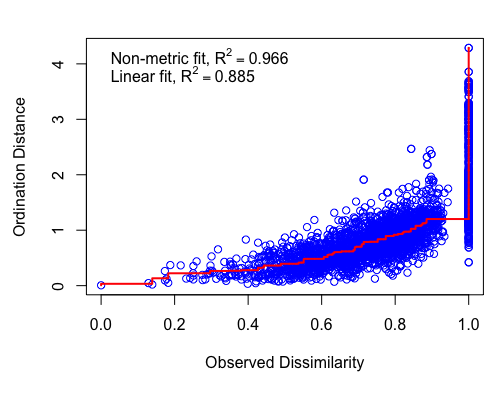


**S4 Fig. 2.** Shepard diagram of ordination fit for beetle dissimilarity matrix.
