# Supplementary figures and images for: Ground-dwelling arthropods of pinyon-juniper woodlands: Arthropod community patterns are driven by climate and overall plant productivity, not host tree species
Source: PLoS One. 2020 Aug 26;15(8):e0238219. doi: 10.1371/journal.pone.0238219 (PMC7449382; doi:10.1371/journal.pone.0238219)

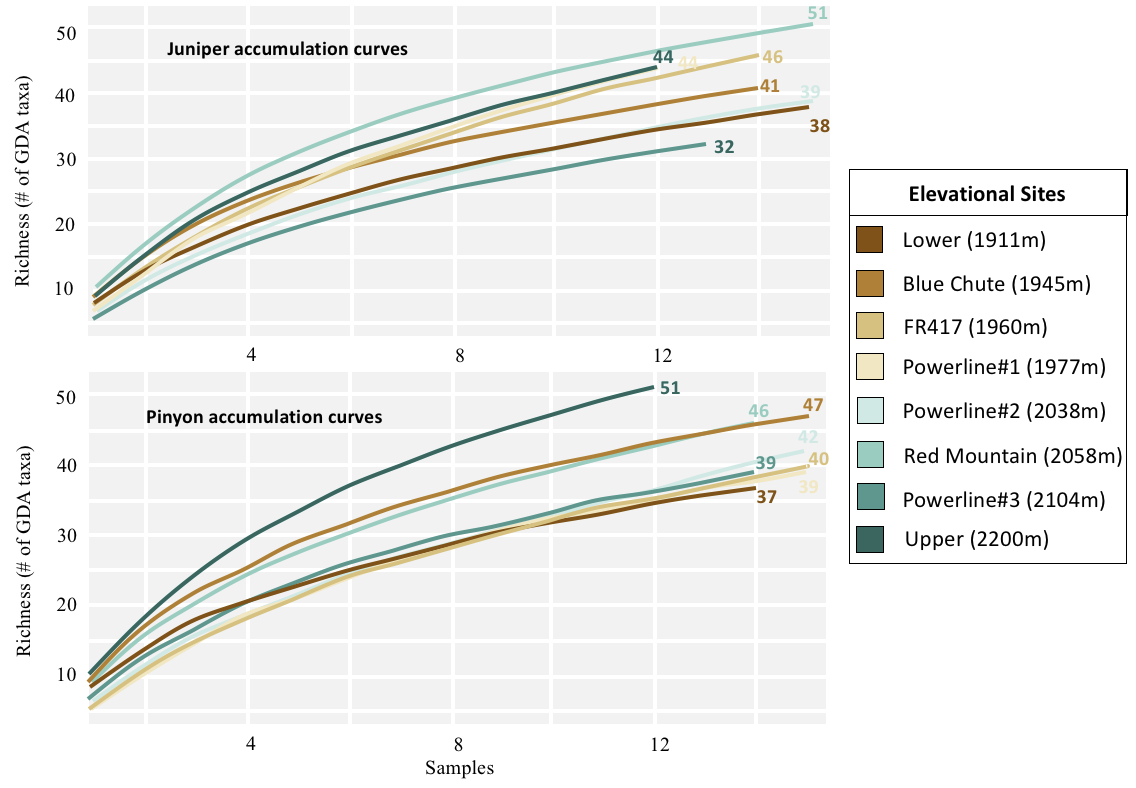

Supplement: S1 Fig — Number of unique taxa accumulated during sampling for both tree species (pinyon and juniper) at elevational sites. (TIF) [file pone.0238219.s011.tif]
