# Supplementary material for: Ground-dwelling arthropods of pinyon-juniper woodlands: Arthropod community patterns are driven by climate and overall plant productivity, not host tree species
Source: PLoS One. 2020 Aug 26;15(8):e0238219. doi: 10.1371/journal.pone.0238219 (PMC7449382; doi:10.1371/journal.pone.0238219)
Supplement: Attachment — Submitted filename: Response to Reviewers.docx [file pone.0238219.s013.docx]

Derek Uhey, PhD student	dau9@nau.edu

School of Forestry	Northern Arizona University	303-961-3984

Flagstaff, AZ 86011

Dear Editor,

Below are our responses (in red) to the reviewers comments for the manuscript, “Ground-dwelling Arthropods of Pinyon-Juniper Woodlands: Arthropod Community Patterns are Driven by Climate and Overall Plant Productivity, not Host Tree Species”, catalog number PONE-D-20-11956.

We appreciate the feedback provided from both reviewers and the editor which has strengthened our manuscript. We have accepted all suggestions and revised our manuscript accordingly. All authors have approved the changes.

Thank you for your time and consideration,

Derek Uhey, Corresponding Author

Northern Arizona University

College of Engineering, Forestry and Natural Sciences

200 E. Pine Knoll Dr.

Flagstaff, AZ 86011 USA

Ph: 303-961-3984

Email: [dau9@nau.edu](mailto:dau9@nau.edu)

**Editor Comments:**

The reviewers and I agree that this revised version of the manuscript is much improved from your initial submission. Reviewers 1 and 2 listed a handful of remaining minor concerns that you should address. I also have my own list of editorial or grammatical edits for you to consider (see "Additional Editor Comments", below). If you address all of these comments, then the manuscript should be suitable for publication.

I received another comment from someone other than the three reviewers of record on your submission. To summarize, this individual argued that you should have used a repeated measures ANOVA approach to analyze your data, rather than generalized linear mixed models. I disagree. Repeated measures approaches, at least in this context, tend to violate the sphericity assumption, and GLMMs offer better flexibility. Nevertheless, it may be worth including a brief sentence that explains why you chose to use GLMMs for your tests of richness and abundance. I'll leave that up to you.

We thank the editor for their suggestion and understanding of our choice of statistical method. We have added a brief statement on why we choose the GLMM approach on lines 209-210 of the methods.

Additional Editor Comments (if provided):

Line 38 - suggest inserting a comma after "woodlands"
Line 44 - suggest inserting a comma after "properties (9,10)"
Line 46 - suggest inserting a comma after "pinyons"
Lines 94-95 - the San Francisco Peaks are a mountain range, i.e., there is no singular San Francisco Peak. The listed elevation is for Humphreys Peak, which is the tallest peak in the range.
Line 100 - capitalize "peaks"
Line 102 - "The relative proportion of pinyons and junipers..." - there are two relative proportions, one for pinyons and another for junipers.
Line 169 - replace "collectivity" with "collectively", or maybe "together"
Line 311 - replace "habit" with "habitat"
Line 328 - replace "are" with "may", or delete "be"
Line 354 - insert commas after "gradient" and after "woodlands"
Line 359 - replace "affect" with "affects"
Line 361 - replace "can't" with "cannot"
Line 373 - insert hyphen between "Low" and "elevation"
Line 375 - make "Ponderosa" lower-case
Line 401 - replace "highlight" with "highlighted"

We have accepted all of the above suggestions and thank the editor for these improvements.

**Reviewer #1**: I commend the authors on doing a really thorough job with the revisions. I just noticed a few minor errors/typos that I am listing here. My only other concern is the data availability. The authors say the data is available to download as supplements or from Symbiota Collection of Arthropod Network, but no link is provided leaving the reader to locate the data themselves, which can be cumbersome. The authors included the R output on my request, but without the csv file, it is difficult to replicate their analyses. So, I would encourage the authors to make the data available in a more easily accessible format.

We thank the reviewer and agree data availability can be improved. We have included all relevant CSV files as supplements (in zip file with R-script) and improved our R-script (S2 File). Unfortunately, our records in Symbiota Collection of Arthropod Network are only searchable by specimen label information. The search platform is found at the given link. We also include links to photographed specimens at bugguide.net with url #s in S6 Table. With the changes to our S2 file, along with our many supplemental materials, we believe our data is easily available.

And here are the other minor edits:

1. Line 263, I think it should say "a significant indicator" instead of "significant indicators";

2. Line 296: Table 2 does not show that NDVI is a significant predictor of ant richness, so I think the authors should remove NDVI from this sentence;

3. Line 311, "habitat" instead of "habit";

4. Line 328: these is an extra "be";

5. Line 330, "exacerbated" instead of "exasperated"

We have revised our manuscript and accepted the reviewer’s suggested line comments.

**Reviewer #2:** The authors have greatly improved the manuscript in this draft, and they have adequately responded to the majority of my previous comments. I do still have a few concerns, but I think they should be easy to address.

First, I am still not satisfied with the handling of ant abundances. I realize it seems like I might be harping on this, but it really is a difficult quantity to measure. The authors run GLMMs for abundance of 1) all GDA, 2) only ants, and 3) only beetles. In Table 2, the estimates for all GDAs and for ants are quite similar, which makes sense because 76% of the individuals were ants. While ants are certainly a very abundant and important component of the GDA community, the observed abundances are not representative given the issues with pitfall traps, particularly with regards to samples along gradients. For example, Camponotus vicinus is not 250x as abundant under tree 135 compared to tree 166, but that is the data going into the model.

Ant workers collected in pitfall traps simply do not represent either colony abundance or worker abundance, and using worker counts as estimates of abundance will (frankly and unfortunately) result in nonsense. Using the worker abundances from pitfall traps directly just does not give the kind of information needed to estimate abundances. My suggestion would be to instead limit the abundance GLMMs to non-ant GDAs, and show results for 'Beetle abundance' and 'Other GDA abundance', where 'other' is non-ant non-beetle taxa. This is only an issue for abundances - richness should be unaffected.

We thank the reviewer for their concerns. The accuracy of estimating ant-abundances via pit-traps is certainly debatable, given we found no significant patterns with ant or GDA abundance, we agree our non-results should be accompanied with more discussion of potential methodological bias. We believe it is still important to report our non-results on the abundance of ants and over-all GDAs but have added discussion on the short-comings of pit-traps for estimating abundance on lines 415-420. Further, we have added the ‘other’ group to our analysis as suggested by the reviewer.

Second, unless time or computing power are limiting factors, it is generally preferable to fit all possible models and compare using AICc rather than using backwards stepwise selection (208-209). However, I think there must be something missing or I am misunderstanding the analyses, as I cannot find any GLMM results for subsets of the predictor variables representing the final models. If it is the former, the results should be included and I would contend that Table 2 should only show the final models. If it is the latter, this section and the results perhaps need some clarifying.

We thank the reviewer for their suggestion and have updated our methods and results to strengthen our GLMM results. We fit all possible models for each GDA response variable and include AICc comparisons in S3 Table. Our methods are updated to reflect this on lines 207-221. We have taken the reviewer suggestion to have table 2 only include final models.

A final minor suggestion for improved reproducibility the future: R notebooks (in RStudio) are a really nice way to provide the code and output. PDFs can be generated easily, with text, code, and output inline without the additional characters that come from pasting into a .docx file (S2). In my opinion, it makes it easier for readers, for anyone interested in trying/adapting your code, and even for the authors.

We are grateful for this suggestion and have included an R-notebook output in our S2 files along with CSV datasheets.

\# Line items
41: 'genus'

Revised.

46-49: I still feel like this needs more qualification or justification... Maybe first rephrase to state the differences found in the bird studies, or even that they found a difference, and then qualify the second sentence with something like 'if other taxa show similar microhabitat specialization, ...'

We have revised these sentences as suggested by reviewer.

200-211: Is it correct that 'abundance' for ants here refers to the number of workers? For clarity, it would be worth stating below (230-232) that incidence was used *only* in the similarity matrices (e.g., "In contrast to the above analyses which directly used the number of ant workers collected...").

We have clarified that use of incidence-based ant abundance was only in similarity matrices for ordination analysis.

Reviewer #3: (No Response)
